# Risk factors for previously unknown methicillin-resistant *Staphylococcus aureus* (MRSA) carriage on admission to 13 surgical wards in Europe

**DOI:** 10.1186/1753-6561-5-S6-O85

**Published:** 2011-06-29

**Authors:** A Pan, A Lee, B Cooper, A Chalfine, G Daikos, S Garilli, S Malhotra-Kumar, JA Martinez, A Patroni, S Harbarth, MOSAR-04 Study Team

**Affiliations:** 1 Istituti Ospitalieri, Cremona, Italy; 2 University of Geneva Hospitals and Faculty of Medicine, Geneva, Switzerland; 3 Mahidol-Oxford Tropical Medicine Research Unit, Bangkok, Thailand; 4 Groupe Hospitalier Paris Saint-Joseph, Paris, France; 5 Laiko General Hospital, Athens, Greece; 6 Universiteit Antwerpen, Antwerp, Belgium; 7 Hospital Clinic de Barcelona, Barcelona, Spain; 8 Ospedale di Esine, Esine, Italy

## Introduction / objectives

MRSA carriers admitted to surgical wards may pose both clinical and epidemiological problems. We performed a prospective, observational cohort study of patients screened for MRSA on admission to 13 surgical wards in 4 European hospitals, to identify risk factors of previously unknown MRSA carriage and to define a common predictive rule.

## Methods

Multivariate logistic regression models were used to predict probabilities of MRSA colonization on admission based on patient characteristics. A scoring system was defined based on odds ratio results. The c-statistic was calculated to evaluate several models.

## Results

We enrolled 2901 patients, of whom 111 (3.8%) were unknown MRSA carriers on admission. We identified 7 independent risk factors associated for newly identified MRSA carriage on admission: urinary catheter, nursing home residency, chronic skin disease, wounds, recent hospitalization, diabetes, and age ≥70 years. No risk factor was common to all 4 centres. The overall prediction rule with a lower cut-off had 87% sensitivity and 32% specificity, while values for a higher cut-off were 40% and 89%, respectively. Local predictive rules performed slightly better: 56% sensitivity and 96% specificity for Barcelona. The c-statistic for the model including all centres was 0.64, indicating limited predictive power of the common model.

## Conclusion

Risk factors for unknown MRSA carriage vary substantially between surgical wards across Europe. A common predictive rule is of limited clinical value.

## Disclosure of interest

None declared.

